# Experimental and Theoretical Studies on Sustainable Synthesis of Gold Sol Displaying Dichroic Effect

**DOI:** 10.3390/nano11010236

**Published:** 2021-01-18

**Authors:** Anshuman Jakhmola, Raffaele Vecchione, Valentina Onesto, Francesco Gentile, Maurizio Celentano, Paolo Antonio Netti

**Affiliations:** 1 Istituto Italiano di Tecnologia, IIT@CRIB, Largo Barsanti e Matteucci 53, 80125 Napoli, Italy; m.celentano@qub.ac.uk (M.C.); nettipa@unina.it (P.A.N.); 2 Centro di Ricerca Interdipartimentale sui Biomateriali CRIB, Università di Napoli Federico II, Piazzale Tecchio 80, 80125 Napoli, Italy; 3 CNR NANOTEC—Institute of Nanotechnology c/o Campus Ecotekne, via Monteroni, 73100 Lecce, Italy; valentina.onesto@nanotec.cnr.it; 4 Department of Electric Engineering and Information Technology, University Federico II, 80125 Naples, Italy; 5 Department of Experimental and Clinical Medicine, University Magna Graecia, 88100 Catanzaro, Italy; 6 School of Chemistry and Chemical Engineering, University Belfast, David Keir Building, 39-123 Stranmillis Rd, Belfast BT9 5AG, Northern Ireland, UK; 7 Dipartimento di Ingegneria Chimica, dei Materiali e della Produzione Industriale, Università di Napoli Federico II, Piazzale Tecchio 80, 80125 Napoli, Italy

**Keywords:** gold nanoparticles, dichroic gold sol, gold nanoplates, diffusion limited aggregation, energy optimization, mathematical models

## Abstract

Gold nanoparticles depending on their shape and mixtures of multiple shapes can exhibit peculiar optical properties, including the dichroic effect typical of the Lycurgus cup, which has puzzled scientists for a long time. Such optical properties have been recently exploited in several fields such as paint technology, sensors, dichroic polarizers, display (LCD) devices, laser applications, solar cells and photothermal therapy among others. In this article, we have demonstrated a simple room temperature one-pot synthesis of gold sol displaying a dichroic effect using a slow reduction protocol involving only trisodium citrate as a reducing agent. We found that the dichroic gold sol can be easily formed at room temperature by reducing gold salt by trisodium citrate below a certain critical concentration. The sol displayed an orangish-brown color in scattered/reflected light and violet/blue/indigo/purple/red/pink in transmitted light, depending on the experimental conditions. With minor changes such as the introduction of a third molecule or replacing a small amount of water in the reaction mixture with ethanol, the color of the gold sol under transmitted light changed and a variety of shades of red, pink, cobalt blue, violet, magenta and purple were obtained. The main advantage of the proposed method lies in its simplicity, which involves the identification of the right ratio of the reactants, and simple mixing of reactants at room temperature with no other requirements. TEM micrographs displayed the formation of two main types of particles viz. single crystal gold nanoplates and polycrystalline faceted polyhedron nanoparticles. The mechanism of growth of the nanoplates and faceted polyhedron particles have been described by an enhanced diffusion limited aggregation numerical scheme, where it was assumed that both trisodium citrate and the gold ions in solution undergo a stochastic Brownian motion, and that the evolution of the entire system is regulated by a principle of energy minimization. The predictions of the model matched with the experiments with a good accuracy, indicating that the initial hypothesis is correct.

## 1. Introduction

The outstanding optical properties of colloidal solutions of coinage metals have been known to mankind since ancient times [1,2]. Metal nanoparticles, particularly gold, silver and copper, absorb and scatter light very efficiently; this results in many types of vibrant colors [3,4]. These optical properties of metal nanoparticles were exploited by artists for centuries and were often used to stain glass and ceramics. One such ancient and beautiful color obtained was “Purple of Cassius”, discovered by Andreas Cassius in 1685, which contained gold nanoparticles and oxides of tin [5]. Modern scientific evaluation of colloidal gold began much later with the pioneering work of Michael Faraday in the 1850s, when he accidentally created a ruby red color with gold salts [6]. He found out that the color was actually due to the miniature gold particles, when he studied their light-scattering properties, which are now collectively called the Faraday–Tyndall effect [7]. It is now well known that the color of noble metal nanoparticles is dependent on their properties such as size, shape, surface chemistry, or aggregation state. An impressive example of the optical effect of metal nanoparticles is the glass piece from the 4th century, the mysterious Lycurgus cup which shines red in transmitted light and green in normal light [8]. The mystery of this puzzle of changing color (dichroic effect) in transmitted and scattered light could be solved only in 1990 when scientists discovered gold, silver and copper nanoparticles in the glassy matrix [9]. This dichroic behavior of gold nanoparticles arises due to simultaneous transmission and scattering of light by gold nanoparticles [10,11,12,13]. El-Sayed group studied the absorption and scattering properties of gold nanoparticles of different diameters (20, 40, and 80 nm). They found out that gold nanoparticles in the size range of 20 and 40 nm hardly displayed any scattering phenomenon, but those of 80 nm displayed a notable scattering phenomenon, which was five orders of magnitude higher than that of fluorescein [14]. In modern times, a variety of the colloidal form of gold has been developed whose transmitted colors depends on the morphology of the particles. For example, the colloidal solution of very small gold nanoparticles with particle sizes less than 3 nm display a light brownish transmitted color [15]. This transmitted color changes to other colors when the particle morphology changes—for example, wine red for spherical, (5–20 nm) [16,17], blue for nanoflowers (50–100 nm) [18], reddish-brown [19], green [20] and orange for gold nanorods of different aspect ratios or gold nanoflowers [20,21] and finally to black when particles are in the form of nanowire networks or twisted gold nanorods [22,23]. All of these morphologies have attracted great scientific attention due to their wide range of applications in various engineering and technological fields such as laser applications [24], solar cells [25], photothermal therapy [26], drug delivery [27], X-ray computed tomography [28], sensors [29,30], dichroic polarizer [31,32], display (LCD) devices [31,33] and paint technology [34,35]. In general, establishing control over the sizes and shapes of the nanoparticles has prompted nanocrystal research to explore new methods to finely control the architecture of the nanocrystals for various scientific and technological applications [36]. However, one-step room temperature protocols that enable convenient production of colloidal nanocrystals are still scarce [23,37]. Additionally, environmental concerns have motivated the search for alternative ecofriendly methods for the synthesis of gold nanomaterials, which are in high demand in almost all scientific and technological fields. The citrate-based reduction of gold salts pioneered by Turkevich et al. in 1951 is still one of the most preferred and most intensively studied method, requiring only two starting materials viz. aqueous solutions of sodium citrate and gold(III) chloride and some sources of energy (generally heating at boiling water temperatures) [38]. The basic and most stable morphology of the particles obtained by this method is the spherical shape with diameters ranging from 10 to 30 nm [39]. The diameters could be further increased to 300 nm by the seeded growth approach and by carefully adjusting the reaction conditions to inhibit any secondary nucleation [40,41]. More recently, a variety of other shapes such as quasi-spherical [18], nanoplates [42], nanowire network (NWN) [22], nanoflowers [18], ultrasmall gold nanoparticles [16,17], twisted gold nanorods [23] and colloidal gold displaying dichroic effect [10,11,12,13,43] have also been obtained by using citrate as a major reducing agent.

To the best of our knowledge, the citrate-based protocol we are proposing here represents the easiest method to synthesize colloidal gold sol displaying dichroic effects without the use of any seed particles [13] or heating above room temperature [11,12]. Previously, we described how gold nanowire networks and gold foam could be prepared at room temperature by the citrate reduction method [22] and how morphology could be manipulated at room temperature by adding a third molecule to the reaction system [16,17,18,23]. In the present paper, we have further extended our work to the formation of dichroic sol displaying various transmitted colors by tuning the experimental protocol using the classical citrate method. Gold sol displaying a dichroic effect has already been reported in the literature by different methods, but more stringent conditions have been used to develop and design them [44]. For example, Liu and coworkers synthesized gold sol displaying a dichroic effect using a multistep protocol, which first involved the synthesis of classical red-colored gold sol which acted as gold seeds. These seeds were then processed in various ways to form dichroic sol of different colors [13]. Saggiomo and his group recently published two articles where they were able to synthesize gold sol displaying dichroic effects by boiling gold salt in the presence of sodium citrate [11,12]. They were able to replicate the dichroic effects of the Lycurgus cup by this method by using gold and silver nanoparticles. Finally, we have demonstrated a numerical scheme, partially based on a diffusion-limited aggregation (DLA) scheme, capable of reproducing the growth of gold nanoparticles. The model generates aggregates of a large amount of particles starting from an initial seed and assumes that (i) the motion of ions in a solution is a random walk, and that (ii) particle deposition at each step of the process optimizes the energy of the system. Using the model, we obtained several different numerical aggregates whose shapes and growth dynamics match with the experiments with very high accuracy.

## 2. Materials and Methods

### 2.1. Materials

Gold (III) chloride trihydrate (99.9%), trisodium citrate dihydrate, polyvinyl alcohol (PVA) (31,000–50,000), polyvinylpyrrolidone (PVP) (10,000), dextran (70,000), were purchased from Sigma Aldrich (Milan, Italy) and were used as such. Milli-Q water was used in all the experiments.

### 2.2. Synthesis of Dichroic Sol

The basic principle of synthesis methodology was based on the ecofriendly protocol already developed in our lab with which we could easily synthesize gold nanowire networks and gold foam [22]. In summary, stock solutions of chloroauric acid (4.0 mM), trisodium citrate (38.8 mM) were prepared in milli-Q water. For most of the experiments, we prepared 1 mL of a sample by taking 0.5 mL of stock solution of chloroauric acid in a 1.5 mL microcentrifuge tube followed by the addition of 0.45 mL of milliQ water and 50 µL stock solution of trisodium citrate. We also added micromolar amounts of PVA, PVP, dextran, ethanol, etc., in the protocol while maintaining the molar ratio R = 0.97 throughout. The stock solutions of PVA, PVP, and dextran were prepared by dissolving 10 mg of these materials in 1 mL of milliQ water. The final concentrations were C_HAuCl4_ = 2.0 mM and C_citrate_ = 1.94 mM with a molar ratio R = C_citrate_/C_HAuCl4_ equal to 0.97.

### 2.3. Characterization

Gold nanoparticles were mostly characterized by Cryo-TEM and UV–Vis Spectroscopy. UV–Vis Spectroscopy was performed on a Cary^®^ 100 UV–Vis, Varian (Agilent Technologies, Milan, Italy) and 1 and/or 2 mm quartz cells were used to measure the absorption spectrum or perform kinetic studies. TEM micrographs and electron diffraction patterns (selected area electron diffraction; SAED) were obtained using a TECNAI G2 F20 Transmission electron microscope (FEI company, Eindhoven, The Netherland) equipped with a Schottky field emission gun operating at an acceleration voltage of 200 kV and recorded at Ultrascan (Gatan, Pleasanton, CA, USA) CCD camera. SEM images and energy dispersive X-ray (EDS) measurements were obtained using FESEM ULTRA-PLUS (Zeiss) Scanning electron microscope (Milan, Italy).For these measurements, a small drop of solution containing gold nanocrystals was spread onto the surface of a silica wafer and was carefully mounted on an aluminum stub covered by carbon tape. No sputter coating was performed on the samples, which were then placed on the sample holder of the microscope and imaged using 10–20 kV accelerating voltage.

### 2.4. Generating Numerical Aggregates

The mechanism of metal growth was reproduced using a diffusion-limited aggregation (DLA) scheme, where randomly displaced nanoparticles stick together to form the final structures. In the simulations, a square lattice was considered as a domain and six initial seeds (i.e., the nucleation sites representing the particle precursor) were chosen. Cellular automata were used to reproduce the displacement of gold ions in the domain prior to deposition: the path of the ions was chaotic, and at each iteration, each ion was displaced randomly in the lattice. Upon contact with the seed, ions in the domain were incorporated into the aggregate according to the sticking probability sp, which describes the rate the aggregate was generated to. For each of the seeds, we conducted simulations with sp=(1, 0.5, 0.1, 0.05) and after 1M of iterations, the final aggregate was formed. In a second set of simulations with the same seeds and sp values, a further condition was introduced. Specifically, a particle could stick to the aggregate with a site-selective probability depending on the coordination number c according to the formula $p_{e}=\left(9-c\right)/8$. c describes how many particles are connected to a specific anchoring point of the aggregate and can assume values between 1 (if the considered particle is located in correspondence of an edge of the aggregate) and 8 (if the considered particle is completely surrounded by other particles). So, if c=1, the probability a particle to stick to the aggregate is $p_{e}=1$.

## 3. Results and Discussion

**Figure 1:**
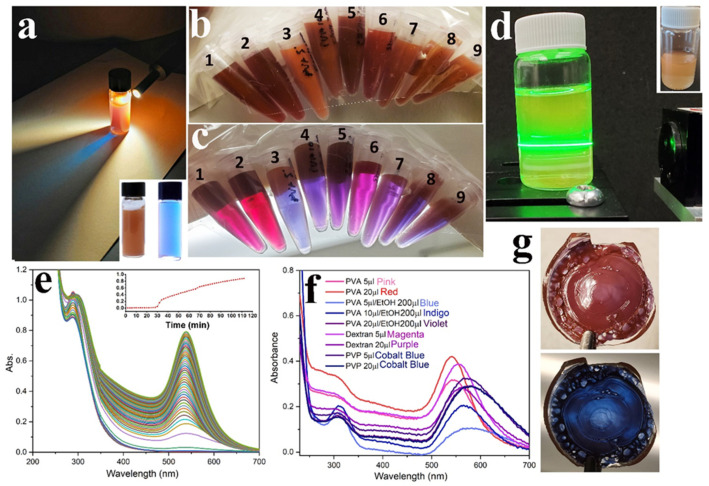
Dichroic gold: (**a**) Dichroic gold synthesized at room temperature by mixing aqueous solution of trisodium citrate and chloroauric acid Au^3+^ 500 µL + trisodium citrate 50 µL + water 450 µL. The color of transmitted light is blue while scatter light is golden-brown. (**b**) Dichromic gold sol prepared by adding a third molecule/solvent in the reaction mixture displaying different shades of golden-brown color in scattered light. Au^3+^ 500 µL + trisodium citrate 50 µL in all samples plus (1) water 450 µL + polyvinyl alcohol (PVA) 5 µL, (2) water 450 µL + PVA 20 µL, (3) water 250 µL + EtOH 200 µL + PVA 5 µL, (4) water 250 µL + EtOH 200 µL + PVA 10 µL, (5) water 250µL + EtOH 200 µL + PVA 20 µL, (6) water 450 µL + Dextran 5 µL, (7) water 450 µL + Dextran 20 µL, (8) water 450 µL + polyvinylpyrrolidone (PVP) 5 µL and (9) water 450 µL + PVP 20 µL. (**c**) Same sol in transmitted light. The colors of transmitted light are (1) pink, (2) red, (3) blue, (4) indigo, (5) violet, (6) magenta, (7) purple, (8) cobalt blue and (9) cobalt blue. (**d**) Dichoric sol displaying a Tyndall effect when green laser beam was passed through it. (**e**) UV–Vis kinetics of dichroic sol; inset evolution and growth of plasmon band with respect to time. (**f**) UV–Vis spectra of different dichroic sols as in (**b**,**c**). (**g**) PVA–dichromic gold composite film on glass surface displaying dichroic effect, the dichroic effect is retained in solid form when dried at 70 °C. C_HAuCl4_ = 2.0 mM, C_citrate_= 1.94 mM with molar ratio R = C_citrate_/C_HAuCl4_ equal to 0.97 in all samples.

**Figure 2:**
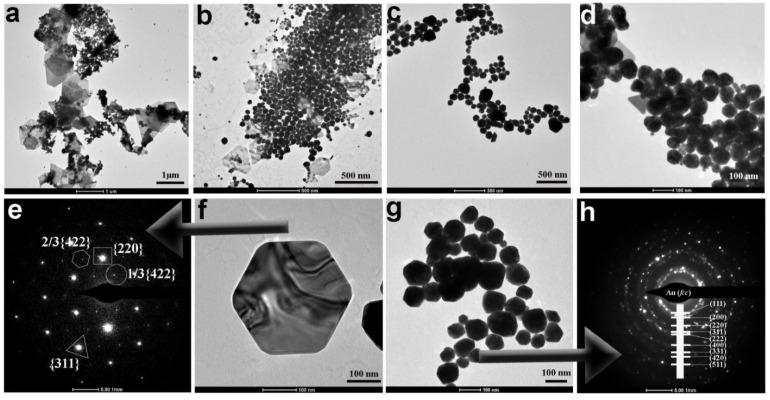
(**a**,**b**) TEM micrographs of dichroic gold at different magnification displaying formation of two different types of nanocrystals, single crystal nanoplates and polycrystalline faceted nanoparticles. (**c**,**d**,**g**) TEM micrographs at different magnification of faceted particles and (**f**) TEM micrograph of a single hexagonal nanoplate of gold. (**e**) SAED pattern hexagonal nanoplate displaying single crystal structure. (**h**) SAED pattern of faceted particle displaying polycrystalline nature and *fcc* structure.

**Figure 3:**
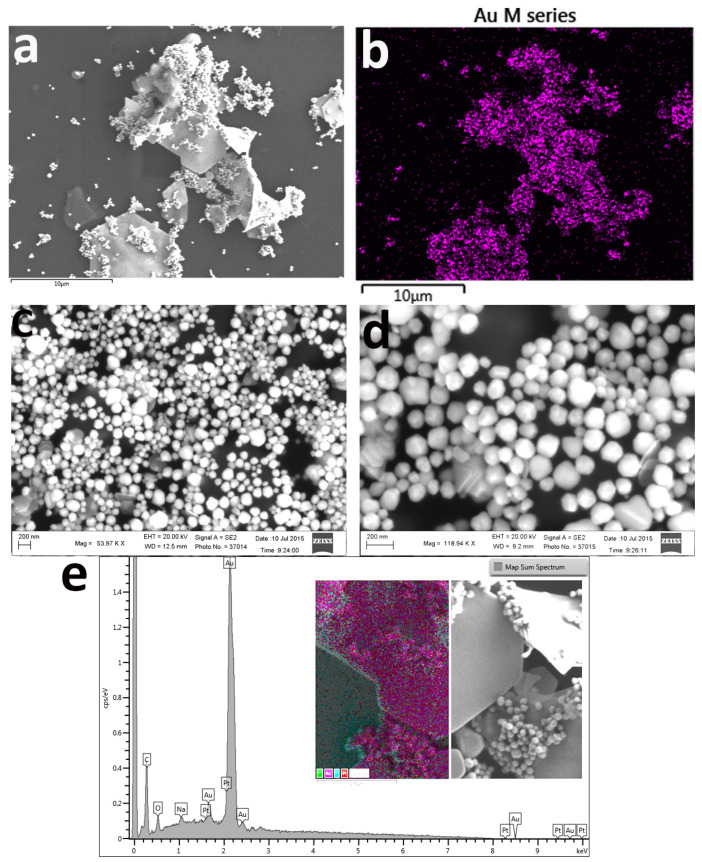
(**a**) SEM images of polyhedral-shaped gold nanoparticles, displaying both nano/micro plates and faceted particles. (**b**) EDX mapping of same image displaying distribution of gold. (**c**,**d**) SEM micrographs of faceted particles at different magnifications. (**e**) EDX of gold nano/micro plates displaying different elements.

The proposed method to produce gold sol displaying a dichroic effect is environment-friendly and further simplifies the Turkevich method [38] as the particles are synthesized under ambient conditions, thus saving unnecessary energy and water wastage during the refluxing process associated with the synthesis at boiling water temperatures. The process involves simply mixing of reactants at a particular ratio and concentration in any sequence followed by solution ageing [22]. Our results indicate that this effect can be easily obtained by playing with the ratios of gold salt and citrate viz. dichroic sol. (ratios between 0.97 and 1.45). Such a product displayed a similar type of optical effect as the famous 4th century Lycurgus Cup [8] made of glass doped with gold and silver nanoparticles, which appeared green and opaque in reflected light and red and transparent in transmitted light. Trisodium citrate is a weak reducing agent; thus, at room temperature, we slowed down the reduction process from a few minutes to hours. The insufficient and scarce amount, as well as weak reduction property of citrate at room temperature, served to control the structure of nanoparticles and induce this dichroic effect. After initial mixing, the yellow-colored solution of auric chloride slowly faded, and a bright golden-brown glittering solution developed after a few hours. This solution displayed blue color in transmitted light and reddish-brown color in scattered light (Figure 1a). This effect was retained when dichroic nanoparticles were embedded in polyvinyl alcohol (PVA) which is one of the most common polymers used for 3D printing and dried to form a thin film (Figure 1g). When this solution was kept in the path of the green laser, Tyndall light scattering was clearly observed (Figure 1d) due to the presence of large polygonal big particles and gold nano/microplates with the size range extending up to micrometer regime, as we will describe in detail later (Figure 2 and Figure 3). This effect is far less prominent in gold nanoparticles with smaller diameters (Supporting Figure S1). Recently, Saggiomo and coworkers [11,12,43] and Liu et al. [13] also demonstrated this dichroic phenomenon using gold salt and citrate, but in both cases boiling water temperature was used. In our work, this phenomenon was observed at room temperature at ratios between 0.97 and 1.45; below this range, the effect became less prominent and at ratios less than 0.39, no reduction could be observed at all. Initial characterization to study the formation of dichroic gold was carried out by following the kinetics using UV–Vis spectroscopy (Figure 1e), which provides important information about the morphology of gold nanoparticles and their surrounding environment. For example, we saw in our previous articles that nanowire networks displayed a flat absorption profile [22] while nanoflowers displayed a broad peak at around ~600 nm, extending up to the near-infrared NIR region [18]. For spherical nanoparticles, a sharp band is observed at around 520 nm, which redshifts as the size increases [16]. The kinetic study of dichroic sol reveals that at time zero (t_0_), the absorption spectrum shows just one peak centered at 290 nm, which is compatible with the electronic spectra of chloroauric ions in combination with chloro-hydroxy species [45]. From Figure 1e, it can be deduced that the synthesis has an induction time of ca 24 min when a plasmonic band centered at around 533 nm emerges. After the increase in absorbance, it displayed a linear trend with time. After around 120 min of reaction, a plasmonic band centered at ca 540 nm was observed, (Figure 1e). Then, we explored the effect of the third molecule and ethanol. In particular, stock solutions of PVA, PVP and dextran were prepared by dissolving 10 mg in 1 mL milli-Q water. A specific amount of this solution was then added to the reaction mixture of gold salt and citrate and the reaction was allowed to proceed as before. We observed an interesting phenomenon: when we added a third molecule and/or ethanol in the reaction mixture, it produced only a minor effect in the color of scattered light (Figure 1b), but the color of transmitted light changed completely (Figure 1c). For example, when PVA was added to the sol (5 µL and 20 µL) (while keeping the total volume of the reaction mixture at 1ml, see experimental), a pink and red color was obtained (Figure 1c). When ethanol was also added along with PVA the following color in transmitted light were obtained: PVA 5 µL + EtOH 200 µL = blue, PVA 10 µL + EtOH 200 µL = indigo, PVA 20 µL + EtOH = 200 µL violet, dextran 5 µL = magenta, dextran 20 µL = purple, PVP 5 and 20 µL = cobalt blue while keeping the concentration of gold salt and citrate constant as before (Figure 1c). The different shades of blue, purple and violet colors can be justified with the presence of large nanoparticles as well as aggregates of different shapes and sizes [46]. This was confirmed in our case by morphological analysis, as reported later (Figure 1f and Supporting Figure S2).

### 3.1. Morphological Characterization

Regarding the samples with no changes to the protocol, TEM and SEM micrographs (Figure 2 and Figure 3) confirmed the structure and revealed the formation of gold nano/microplates together with multifaceted polyhedron nanoparticles, a structure similar to that obtained by reducing gold in deep eutectic solvents [47]. Other shapes including gold nanoparticles, nanoprisms, hexagonal particles, needle-shaped particles, nanorods, and nanosheets among many others depend on the reaction conditions (i.e., addition of a third molecule in the system). The combined optical effect of all these large structures resulted in the peculiar dichroic optical effect of the solution, as the dichroic effect was not seen in solutions containing a majority of gold nanoplates [47]. The selected area electron diffraction (SAED) patterns displayed two different crystallinity; for example, the faceted polyhedron particles were polycrystalline in nature and a ring pattern corresponding to face-centered cubic (*fcc*) structure was obtained for them, while for the nanosheet structures a series of diffraction spots with a six-fold rotational symmetry was obtained (Figure 2e). The latter hexagonal diffraction spots indicated that the nanosheets were single crystalline in nature. The spots could be indexed to the {311}, {220} planes of *fcc* gold and to the formally forbidden 1/3{422} reflections (Figure 2e) [48]. The energy-dispersive X-ray (EDX) analysis and mapping of nanoplates illustrated the purity of the gold, with spectra showing a strong Au signal together with minor peaks of C, Na and O (Figure 3d).

For the samples corresponding to the basic protocol with small amount of PVA, the TEM micrographs of dichroic sol displayed big irregular shaped faceted polyhedron particles(responsible for golden brown scattering light) together with small spherical particles (~10 nm) responsible for the peculiar red color in transmitted light (Supporting Figure S2a,a’). For all other sols displaying pink, violet and indigo color in transmitted light (Figure 1c), a variety of particles of different morphologies could be observed (Supporting Figure S2). The UV–Vis spectra of all these morphologies displaying different transmitted colors are presented in Figure 1f and display broad plasmon bands between 540 and 650 nm corresponding to large particles.

### 3.2. Mechanism of Formation of Gold Nanoplates and Faceted Polyhedron Nanoparticles and Their Association with Dichroic Effect

**Figure 4:**
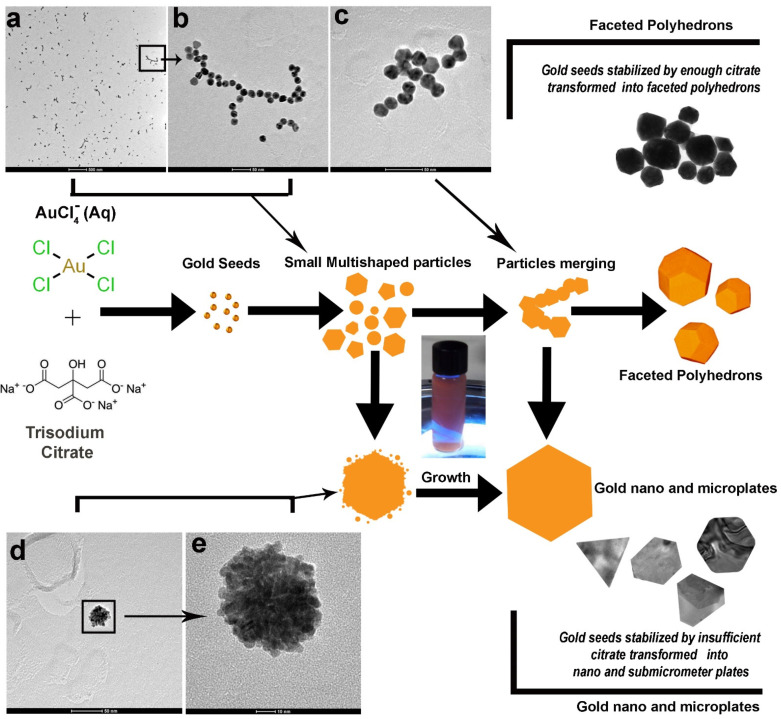
TEM images of intermediates captured during course of reaction displaying (**a**–**c**) aggregates of 10–15 nm gold nanoparticles and (**d**,**e**) displaying growth of small 10–15 nm particles probably by atom-by-atom reduction on the surface. Scheme for the formation of gold nano/micro plates and faced polyhedron nanoparticles as seen in TEM images of intermediates is also presented.

In both TEM and SEM micrographs (Figure 2 and Figure 3), we see the formation of two entirely different types of particles with different crystal structures: big single crystalline nanoplates/microplates and the polycrystalline faceted nanoparticles. The mechanism for the development of nano/microplates is still in debate after their discovery in the 1940s [49]. It is generally believed that Au nanoplates are formed by aggregation and recrystallization of primary particles along a particular crystal plane. This phenomenon occurs because the surfactant molecules interact differently and bind strongly along one plane, thus blocking it and preventing it from growing [48]. This results in the accelerated growth along twin planes, {111} twin planes parallel to each other and along the edges of the plates, thus leading to the formation of nanoplates [50]. However, the more accepted model describes the formation of nanoplates via atom-by-atom reduction on the surface of the gold. To confirm which theory is valid in our case, we carried out TEM analysis during the formation of particles (Figure 4). We found evidence of aggregation of small quasi-spherical nanoparticles (Figure 4a–c) as well as the growth of plate-like particles via atom-by-atom reduction (Figure 4d,e). In addition to nano- and microplates, many 3D faceted polyhedron particles were also formed which we believe was due to multi-twin configurations in early stages, resulting in favor of large faceted 3D polyhedron nanoparticles.

Based on these observations we think that, two major factors work together. The first factor is the relatively low amount of citrate in the reaction mixture as compared to the Turkevich protocol [51] and the second factor is the room temperature due to which the reduction process is further suppressed. Due to these limiting factors, it is obvious that very few gold nuclei will be formed in the reaction mixture. Nucleation represents the very first step in the growth process [52]. Although tremendous efforts have been made to study and understand this process, due to their extremely small size, only limited success has been attained to date. It is well-known that weak reducing agents like citrate, ascorbic acid etc. gives rise to less number of nuclei as compared to strong reducing agents like sodium borohydride which has higher nucleation ability and favors formation of small nanoparticles [53]. These gold nuclei now act as points of growth and form clusters by adsorption of free AuCl^4-^ ions on their surfaces followed by their slow reduction due to poor reducing capabilities of citrate at room temperature. These clusters then give rise to seeds, which hold a key position in bridging nuclei and nanocrystals. During the synthesis and growth process, these seeds, which are stabilized by adequate amount of citrate molecules, result in the formation of large faceted structures, while those seeds which are stabilized by the inadequate and limited amount of citrate result in the formation of nano- and microplates, which, therefore, coexist in a typical synthesis, as in our case (Figure 4) [54]. From detailed kinetic studies, we can conclude that induction time can be associated with the time required for the accumulation of growing gold species to reach the critical concentration necessary to produce stable nuclei. After this induction period, the system passes through a second phase (10 min) in which nucleation and growth occur simultaneously. The absorbance maximum of the plasmonic band grows in linearly and does not reach a plateau in the observed reaction time. The hypochromic shift of the precursor peak from 290 to 302 nm could be related to the precursor consumption in the induction phase. Although AuCl^4-^ increases the attractive forces between two gold nanoparticles, due to the low number of nuclei in solution, the probability of collision of randomly moving particles through Brownian motion is reduced and this may prevent fusion between adjacent particles and consequent unidirectional growth (as seen at higher R values) [22]. Citrate is a weak reducing agent at room temperature and binds weakly on the surface of gold nanoparticles [55]. In addition, we have a relatively low and insufficient amount of citrate in the reaction system as compared to other protocols involving citrate as reducing agent [56]. All these factors act together and result in inefficient capping and stabilization of the particles so that multiple types of faceted shapes are formed depending on which points on the surface of nuclei are shielded by citrate (Figure 2 and Figure 3). The low temperature also favors slow crystallization which promotes the formation of single crystals and their homogenous growth to sheet type structures (Figure 2 and Figure 3).

Light scattering is typically very sensitive to the aggregation state of the sample, with the scattering contribution increasing as the particles aggregate to a greater extent. For example, the optical properties of metal nanoparticles change when particles aggregate and the conduction electrons near each particle surface become delocalized and are shared amongst neighboring particles [57]. When this occurs, the localized surface plasmon resonance peak (LSPR) shifts to lower energies, causing the absorption and scattering peaks to redshift to longer wavelengths [58]. The existence of light scattering and blue shifting could indicate an initial aggregated state for the investigated systems and may be related to an initial poor stabilization of single gold nanoparticles.

### 3.3. pH Monitoring

Both nucleation and growth phases are heavily dependent on pH and its variation throughout the process because citrate ions and chloroauric ions experience pH-dependent speciation (Tables S1 and S2, Supporting Information). These different ionic species have slightly different redox thermodynamics and kinetics, giving rise to speciation-dependent nanoparticle nucleation and growth.

It is known that hydrogen ions are produced from the reaction between gold (III) species and citrate species, thus the redox reaction should clearly be predicted by pH variations over time. Therefore, we have conducted electrochemical experiments to monitor pH during the synthesis of gold nanoparticles. Initial pH values for the two starting solutions for each molecular ratio are reported in Table S3 (Supporting Information).

**Figure 5:**
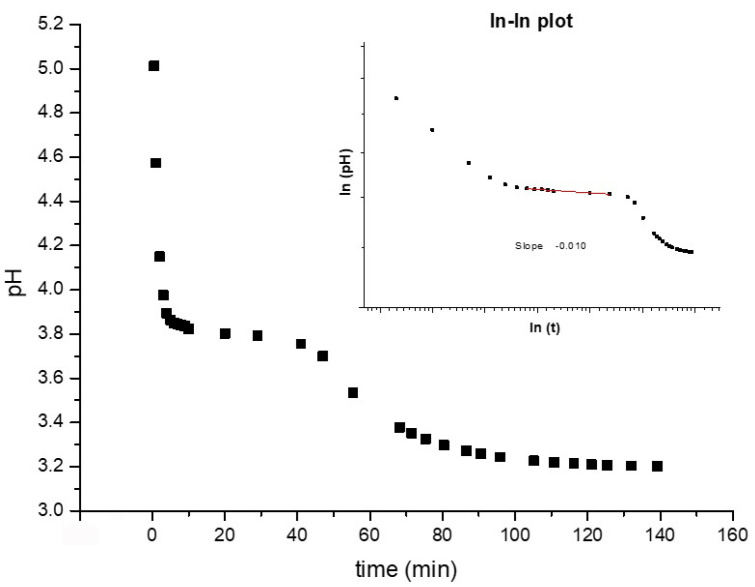
pH variation monitored over time during the dichroic sol synthesis (inset shows the corresponding ln-ln plot).

The chloroauric acid solution was added to the citrate solution and mixed for 5 s, as reported in the experimental section, and then the pH variations were monitored over time (Figure 5). Once chloroauric acid is added to the citrate solution, the pH decreases rapidly down to pH ≈ 3.82 within 10 min. After this time, the pH of the solution changes to ≈ 3.75 within ≈ 30 min. At this point, the composition of the solution changes drastically, and indeed the dominant species in the solution are chloroauric ions and dihydrogen citrate ions near and below this pH value. After 47 min, a rapid exponential decrease by 0.5 pH units is was until the end of the measurement (from pH ≈ 3.7 to ≈ 3.2). Three characteristic regions can be distinguished in the “pH vs. time” curve in Figure 5. Typically, the pH decreases exponentially in the first region, and the process is completed within the first 10 min after mixing. This feature might come from the quick acid–basic reaction between the strong acidic chloroauric solution and the alkaline citrate solution with a strong buffer capacity. The induction period measured by UV–Vis spectroscopy is larger than 10 min, so it is reasonable to rule out any effect coming from the nucleation and growth on the acid/base reaction. In the second region of the graph, the pH decreases again, but more slowly. The pH decays more rapidly when the molecular ratio is lowered. In the third region, pH quickly decreases once again. Furthermore, how rapidly it falls depends on the molecular ratio between citrate species and Au(III) species.

The behavior observed in the second region of the graph is probably associated with the buffer capacity of the citrate solution, which is opposed to the change in pH due to the redox reaction. The pH in the second region is almost steady and persists for a sufficient amount of time to guarantee the stabilization of the speciation of citrate and gold (III) chloride. In the third region, the trend changes drastically, probably due to the rapid consumption of A^-^ and HA, and consequently, the rapid change in their concentrations is reflected in a sustained lowering or loss of buffer capacity. The first two regions can be reasonably assigned to the induction period, which is ascribed to the initial stage when the nucleation is the predominant mechanism in place, although with slight concomitant nanoparticle growth. In support of this interpretation, the time elapsed until the end of the second region appears to be in good agreement with the behavior shown by the UV–Vis spectra. The pH monitoring also suggests that at the end of the induction period the nanoparticles grow quickly following the nucleation phase (third region). Furthermore, the abrupt variations observed in the third region suggest an activated grow mechanism.

### 3.4. A Diffusion Limited Aggregation Scheme with Energy Optimization for the Synthesis of Multifaceted Nanoparticles

**Figure 6:**
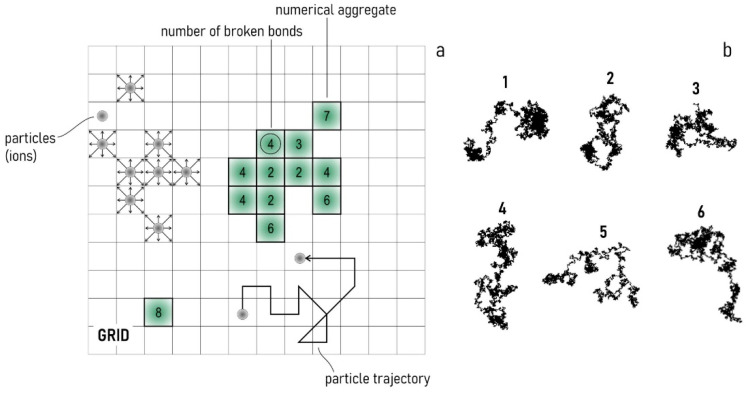
The numerical grid where the process of diffusion and aggregation is simulated. In the grid, the movement of a particle is discretized in finite steps. The aggregate is given by the accumulation of smaller elements on a seed. The probability of adhesion is proportional to the number of broken bonds of each element of the aggregate (**a**). The trajectory of individual particles in the domain is a random walk (**b**).

We used a modified diffusion limited aggregation (DLA) scheme to reproduce the growth of multifaceted nanoparticles. DLA is a classical numerical method that allows the simulation of the aggregation and growth of structures from smaller elements [59,60,61]. In the technique, the random motion of elements is reproduced in a grid, where the pixel size represents the resolution of the system. The formation of complex structures is the result of the accumulation of smaller particles on an initial seed—for this configuration a nucleus of gold is used. Thus, gold ions resulting from the dissociation of gold chloride in solution randomly adhere to the originating nucleus forming an aggregate of a many particles over time (Figure 6a). The entire method is based on the assumption that the motion of small elements in the system is driven by pure diffusion that in turn implies that the trajectory of particles is approximated by a random walk (Figure 6b). Moreover, the adhesion of a particle to the nucleus or the aggregate is regulated by the amount of trisodium citrate in solution: an overabundance of citrate over gold chloride (high R, with $R=Citrate/HAuCl_{4}$) slows down the reaction of synthesis of gold nanoparticles. In the numerical scheme, the effect of R is incorporated by the sticking probability sp, a parameter that can be varied between 0 and 1 [62]. If sp is set to 0, the aggregation process stops. If sp is set to 1, every particle colliding with the aggregate is incorporated by it instantaneously. Intermediate values of sp allow the modulation of the reaction rate of growth of the aggregate. While in classical DLA the process of aggregation depends on the sole trajectory of the particles and the probability of adhesion, in real processes the configuration of a system determines the evolution of that system over time. The shape of a structure at an instant of time influences the accumulation and deposition of additional particles on that structure, and the growth of the aggregate for each successive instant of time. An established result in chemistry is that the formation of single crystals is driven by the minimization of the total interfacial energy γ of a system with a given volume. The interfacial free energy is defined as the energy G required for creating a unit area of new surface A, for fixed temperature (T) and pressure (P) [52]:

(1) $$\gamma ={\frac{\partial G}{\partial A}|}_{T,\;P}.$$

In crystals’ growth, the interfacial free energy may be approximated by the simple formula [63]:

(2) $$\gamma =\frac{1}{2}\;N_{b}\;\epsilon \;\rho_{A},$$

where $N_{b}$ is the number of broken bonds, ϵ is the bond strength and $\rho_{A}$ is the density of surface atoms. Assuming that ϵ and $\rho_{A}$ change slowly with time, the variation of γ is associated with the variation of the sole number of broken bonds, so that optimizing γ is equivalent to keeping $N_{b}$ at a minimum. The numerical scheme that we have developed in this work simulates this effect. For each configuration, we registered the number of broken bonds for each point of the aggregate. A particle surrounded by other particles will have $N_{b}=0$ because all the links of the particle with its neighbors are established. For an isolated particle, $N_{b}=8$. Then, we calculated for that point an energy-based probability $p_{e}\;\sim \;N_{b}$. This categorical definition of $p_{e}$ implies that the larger $N_{b}$, the higher the probability that a colliding particle may adhere to a point of an aggregate because, as a result of the aggregation event, the value of $N_{b}$ would decrease, consistent with spatial and energetic constrains and Equation (1). Thus, in this scheme, the growth dynamics of the numerical aggregate is the result of three separate mechanisms: (i) the random walk of the elemental building blocks in the system, which describes the characteristics of the reducing agent (gold chloride) in solution; (ii) the probability of adhesion of a particle to the aggregate, sp, that describes the action of the moderation agent sodium citrate in solution; (iii) the probability of adhesion of a particle to the aggregate $p_{e}$, which translates a physical principle of energy minimization in mathematical terms. We called this modified version of a classical DLA scheme energy minimization DLA (EM DLA).

#### 3.4.1. Nanoparticle Growth and Morphology and Effect of the Seed Geometry

**Figure 7:**
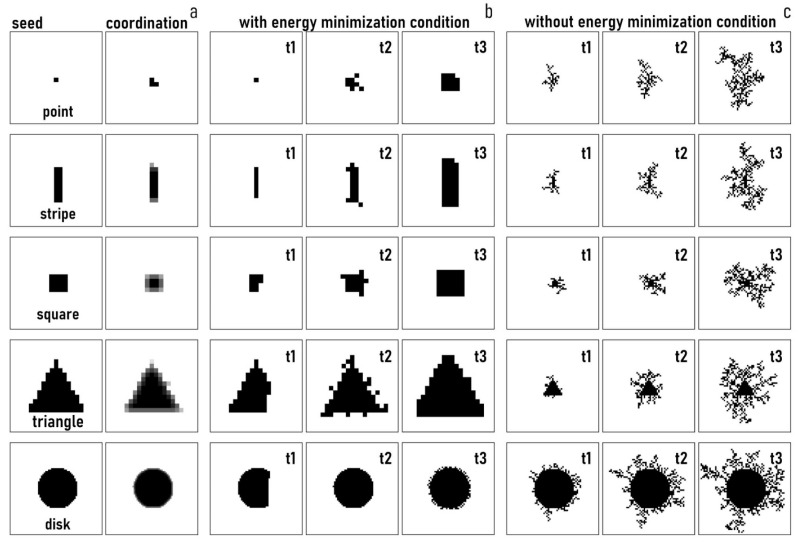
The initial seeds around which the numerical aggregates were formed and coordination number associated with those seeds (**a**). Time evolution of the aggregate for different initial seeds enforcing the energy minimization condition in the diffusion limited aggregation algorithm (**b**). Time evolution of the aggregate for different initial seeds without the energy minimization condition in the diffusion limited aggregation algorithm (**c**).

We used an EM DLA scheme to reproduce the growth of nanocrystals. We launched several different simulations in which the shape of the seed was varied to examine whether the morphology of the final aggregate depends on the geometry of the originating nucleus. We used a point, an elongated, rectangular stripe, a square, a triangle, and a disk as a starting pattern. The raster plot of each of these initial seeds is reported in Figure 7a along with the density plots of their coordination numbers—i.e., $c=8-N_{b}$. Thus, for example, the square with the maximum number of links is found at the center of the pattern, where points are embedded in a matrix of other points and the number of connections (c) is maximized. At the borders, the value of c is lower, and still lower at the corners, where each element of the structure is in contact with other elements for three-eighths of their perimeters and is exposed to air for the remaining five-eighths. We then examined the growth of the aggregates over time of each configuration (Figure 6b). By enforcing the energy minimization condition, we found that the shape of the numerical aggregate was similar at each time of the simulation to that of the originating seed. For the stripe, the square, the triangle and the disk, the shape of the aggregates is a scaled version of the initial nuclei. For the point, the final aggregate is a square, possibly because it is discretized as a squared element in the grid domain. For each configuration, the initial symmetry of the seed was not broken by the process of growth and particle synthesis. The 4− and 2− order rotational symmetry of the square and the rectangle, the 3− order rotational symmetry of the triangle, and the circular symmetry of the disk were maintained throughout the process of formation of the aggregate. An initial anisotropic shape was not blurred into undistinguishable and isotropic shapes because the rules at the basis of the EM DLA scheme are themselves reversible. Remarkably, this implies that the initial information on a structure or a nucleus does not degrade with time. Notably, the same algorithm without the energy minimization condition produces dendritic structures without or with poor correlations with the initial seed (Figure 6e). The numerical scheme that we have developed seems adequate to accurately simulate the formation of nanocrystals and geometric primitives that contain the attributes and behavior of the nanoparticles produced under the experimental conditions described in the rest of the paper, including multifaceted nanoparticles and structures with a variety of different shapes, such as rhombohedral, prismatic and pyramidal.

#### 3.4.2. Dynamics of Nanoparticle Growth and Effect of Sticking Probability

**Figure 8:**
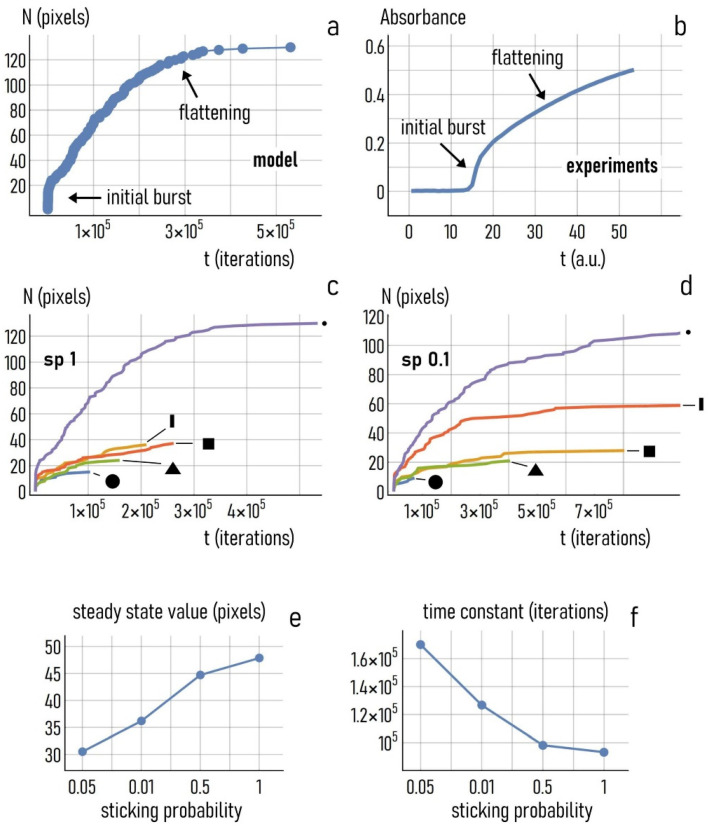
Growth dynamics of a numerical aggregate: for this configuration, the initial seed is a point (**a**). Growth dynamics of the real physical prototype determined as the variation over time of the absorbance peak measured through UV spectroscopy (**b**). Curves of growth of the numerical aggregates for different types of the initial seed and sticking probability *sp* = 1 (**c**). Curves of growth of the numerical aggregates for different types of the initial seed and sticking probability *sp* = 0.1 (**d**). Steady state value of the aggregate mass (**e**) and time to the steady state (**f**) as a function of the sticking probability.

We used data from the simulations to examine how the numerical aggregates grow over time. We report in Figure 8a the mass of the aggregate (i.e., the number of pixels in the aggregates) as a function of the number of iterations for the case of a point as initial seed. We observed that in the early stage of the process the mass rose steeply, with an initial burst followed by an increasing flattening of the curve of growth, which attained a steady state value after approximately $4\times 10^{5}$ iterations. The growth dynamics of the numerical aggregate seem to faithfully follow the dynamics of first order systems, suggesting that this is also the order of the reaction kinetics of the process of particle deposition. Notably, the growth profile of the system inferred from experiments, illustrated in Figure 8b, matches with the model template with very high accuracy. The evolution of the real gold nanocrystals was marked by an initial burst, followed by a smooth deflatation, which seems to drive the system towards a steady state regime, in agreement with the simulation results. Figure 8c reports the growth dynamics of the system for different shapes of the initial seed and a sticking probability sp=1. While the curves of growth exhibit the same trend, there are differences in terms of steady state value and time to steady state, probably because the shape and size of the initial seed affect the way particles accumulate over time. In the case of a point as an initial seed, which is much smaller than the disk, there is more room for the incoming particles to be accommodated around the seed, and the process takes more time. Bringing the sticking probability of adhesion of a particle to the aggregate to sp=0.1 (Figure 8d), we observed that the characteristics of growth also changed. Notably, for this value for sticking probability the differences between different configurations were more marked. We then examined how the dynamics of deposition changed as a function of sp. We launched simulations in which the value of sticking probability was varied between sp=0.05 and sp=1 for different initial seed configurations. For each simulation, we determined the deposition profile. Wanting to approximate the curves of growth with an exponential function of time of the form $N\left(t\right)=N_{s}\left(1-e^{-t/\tau }\right)$, we found after nonlinear fitting of data the solutions for $N_{s}$ and τ reported in Figure 8e,f. $N_{s}$ is the mass of the aggregate reached in the steady state. τ is the time constant, i.e., the time necessary to the system to reach 66% of the final mass. For each sp, values shown in Figure 8e and Figure 7f were averaged over different initial seeds. We observed that the steady state value $N_{s}$ increased from $N_{s\;}\sim \;30$ for sp=0.05 to $N_{s}\;\sim \;48$ for sp=1, with a 60% increment. Differently, in the same interval of sp, the time constant τ decreased from $\tau \;\sim \;1.65\times 10^{5}$ iterations for sp=0.05 to $\tau \;\sim \;1\times 10^{5}$ iterations for sp=1, with a 60% variation similar to that recorded for $N_{s}$. Thus, low values of sticking probability slowed down the entire process of particle deposition. Since sp was varied smoothly between the low (0.05) and high (1) limits of probability range for these tests, results of the simulations indicate that under thermodynamic equilibrium the addition of trisodium citrate to the growth solution may accelerate or hamper the process of growth and the size of the final crystals up to two times.

#### 3.4.3. Shape Analysis of Numerical Aggregates

**Figure 9:**
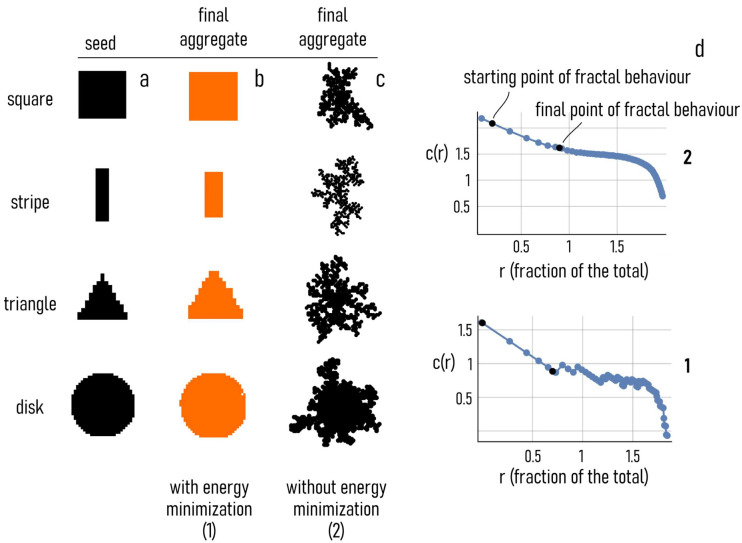
Shape of the initial seeds (**a**) and the numerical aggregates obtained with (**b**) and without (**c**), the energy optimization condition. Density–density correlation function derived for the numerical aggregates (**d**).

**Figure 10:**
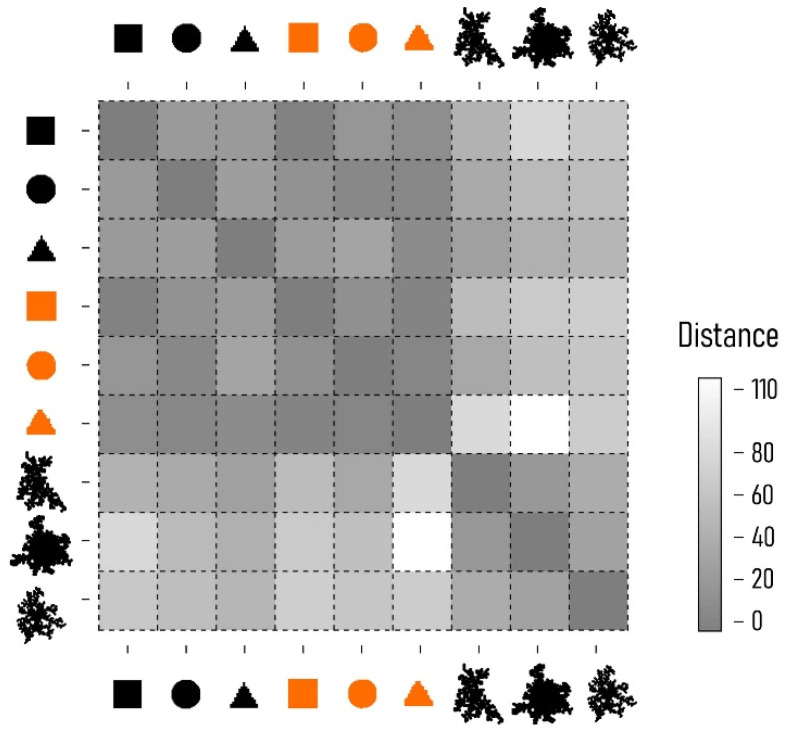
Distance among different numerical aggregates determined canonical warping distance metrics.

Using the energy minimization condition, we observed that the shape of the aggregate at the steady state of the process resembles the morphology of the originating seeds (Figure 9a,b). Differently, without the energy minimization condition the numerical aggregates exhibit a branched and multiscale structure typical of fractals (Figure 9c). We used a density–density correlation function [64] to analyze and compare the internal structure of the aggregates resulting from different algorithms (Figure 9d). The density–density correlation function c(r) describes the information content of an aggregate as a function of the spatial coordinate r. If the slope of c(r) is small (horizontal line in a log–log plot) the information content of the aggregate is distributed across different length scales. Differently, if the slope of c(r) is high (vertical line in a log–log plot), the information content of the aggregate is confined in a limited region of the scale-space. Thus, the slope β can be associated to the fractal dimension of an object as $D_{f}=2-\;\beta$ and the density–density correlation function can be used to gain useful information on the nature of that object. The fractal dimension ($D_{f}$) indicates a change in detail to a change of scale of a pattern—it is a number between 1 and 2. $D_{f}$ describes how much of the structure of an object may be represented in terms of classical geometry [65]. Using the tools of fractal theory, we derived the fractal dimension for the aggregate with (1) and without (2) energy minimization condition as $D_{f}^{1}\;\sim \;1$ and $D_{f}^{2}\;\sim \;1.5$. Results are consistent with the observation that aggregates resulting from an energy optimization condition have ordered structures recalling the shape of the initial seeds and of classical graphical primitives, with low values of fractal dimension. On the contrary, aggregates obtained from classical DLA without the energy minimization condition exhibit higher values of fractal dimension, reflecting a complex geometry, low density and a multiscale behavior. To compare structures quantitatively, we used a sequence-alignment distance. We measured the distance between (i) the initial seeds and the aggregates obtained from (ii) the EM DLA algorithm and (iii) classical DLA (Figure 10). We measured the distance among several different graphical objects using Canonical Warping Distance metrics, which identified the optimal warping path that minimizes the Euclidean distance between two data sequences. In the matrix plot of Figure 10, we represent the distance between different objects in grayscale, such that structure pairs that are similar in shape are characterized by darker tones compared to those shapes with dissimilar internal architectures. The analysis shows that there is a very high correspondence between the geometry of the seeds and the morphology of the aggregates obtained from EM DLA with a defined geometry. At the same time, the correlation between the numerical aggregates resulting from classical DLA with a fractal nature and the seeds is low, consistent with the fractal analysis and the results of the paper.

#### 3.4.4. Comparing Simulations with Experiments

The aim of the simulations was not to reproduce the shape of each of the particles that were formed by experiments exactly, but to develop a method that is capable of generating nanocrystals—i.e., particles with well-defined shapes and some internal correlation, resulting in structures with high symmetries. The technique, which is based on classical DLA, however, departs from the latter in that it introduces an energy optimization principle, described in the Methods and throughout the paper. This does not have any precedent in the literature for the numerical simulation of DLA aggregates. While this test campaign was not focused on optimizing performance, it is useful to put the results in context. The main conclusion suggested from experiments is that the entire process of growth can be divided into regimes. In the early stage of growth, the concentration of gold in solution reaches a critical value leading to the formation of stable nuclei. Once the nuclei have grown past a certain size, they become seeds, with a variety of different configurations, constituting the base for the successive particle synthesis, where the preferential absorption of ions to the aggregate determines particle growth. In this phase, because of the very small density of nuclei in solution, the relatively low amount of citrate, and low temperature, the growth is predominantly unidirectional—i.e., ions adhere to the aggregate along the preferential directions of growth. Unidirectional anisotropic deposition of ions onto the aggregate results in particles with regular shapes, high symmetries and crystal structures. The mathematical model that we have developed simulates the characteristics of real gold nanoparticles with accuracy, especially pertaining to particle shape, particle size and the dynamics of growth.

#### 3.4.5. Particle Shape

**Figure 11:**
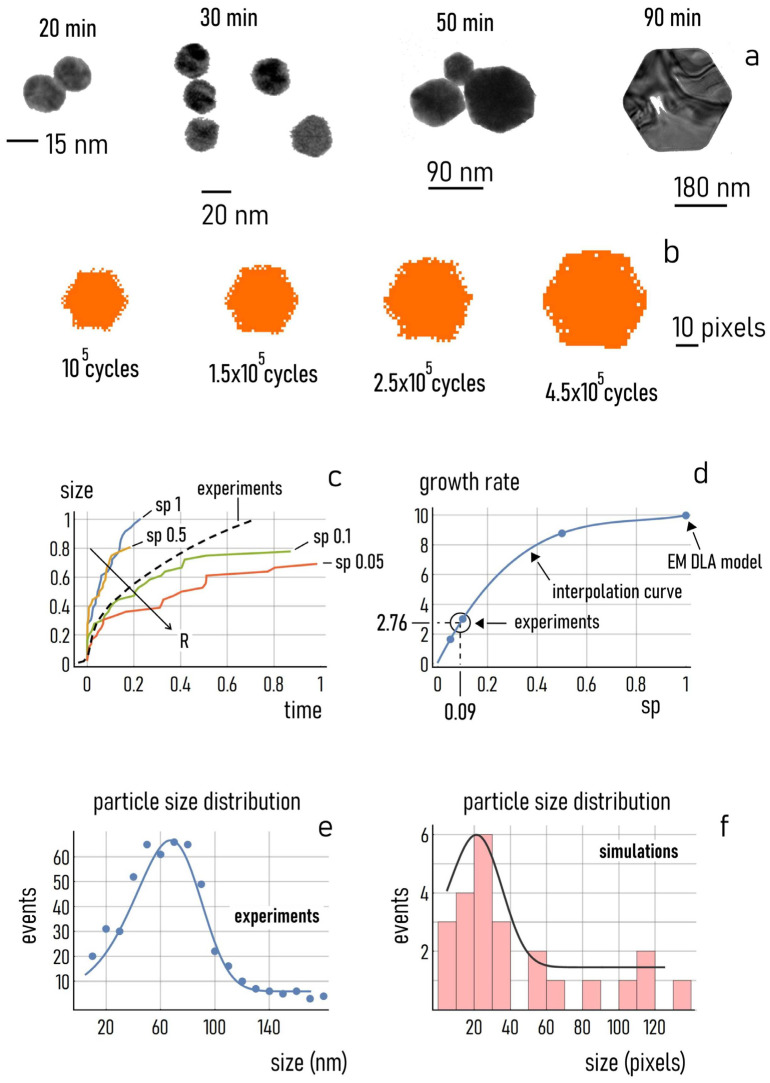
SEM and TEM images of multifaceted gold nanoparticles at different times from the inception of synthesis (**a**). Corresponding numerical aggregates generated by the energy minimization diffusion-limited aggregation (EM DLA) algorithm (**b**). Normalized curves of growth of a numerical aggregate starting from a hexagon as a seed, for different values of the sticking probability, compared to the nondimensional growth profile of real gold nanoparticles (**c**). Rate of growth determined at the early stage of the process for the numerical aggregates at different values of sticking probability: the rate of growth of real nanoparticles is used in the diagram to retrieve the value of sticking probability that leads to the best matching between the predictions of the model template and experimental data (**d**). Particle size distribution determined for the real gold multifaceted nanoparticles (**e**) and the numerical aggregates (**f**); both distributions are skewed to the right.

In the model, the shape of the aggregate shows a very high sensitivity to the configuration of the originating seed. Thus, once the configuration of the seed is determined, the shape of the aggregate will bear a very close resemblance to the originating template at each instant of time. This is analogous to experiments, where the configuration of the nuclei governs particle growth after the initial induction phase. Low citrate content, low temperature, and the absence of possible external elements of interference are among the causes assuring that the energy minimization of the system is maintained throughout particle synthesis. Energy minimization, in turn, leads to unidirectional particle deposition and the formation of gold nanocrystals—i.e., structures with elevated orders, strong internal correlations and high degrees of symmetry. Figure 11a shows TEM and SEM micrographs of hexagonal gold nanoparticles resulting from the process of synthesis at different times. This sequence is compared to the numerical aggregates obtained from the accumulation of smaller elements on an initial hexagonal seed over time (Figure 11b). We observe that: (i) The shape of the numerical aggregates is similar to the morphology of the real nanoparticles for each of the considered instants of time. (ii) The shape of the real nanoparticles becomes more defined over time, possibly because the process of energy optimization of the system, working on statistical scales, requires time to achieve maximum resolution. (iii) Remarkably, the numerical aggregates, while at the early stage of growth show a gross resemblance to the original hexagonal shape, with time evolve into increasingly more well-defined forms, with fewer defects in their internal fabric, similarly to the behavior or real nanoparticles. (iv) The sizes of the real nanoparticles and of the numerical aggregates vary proportionally in the same way in the same deltas of time.

#### 3.4.6. Growth Dynamics and Parameters Identification

The ability of the algorithm to capture the dynamics of particle growth is relevant. In Figure 11c, we report the growth profiles of the numerical aggregates with a hexagon as an initial seed and for different values of sticking probability (sp=0.05, sp=0.1, sp=0.5, sp=1). In reporting data, we have normalized the time scale to the maximum simulation length (i.e., maximum number of cycles), and the size scale to the maximum size of the aggregates, measured among all the simulations examined for this test. In the same diagram, we report the curve of growth of the real gold nanoparticles obtained from UV–Vis measurements of samples. The experimental curve was rescaled between 0 and 1 for direct comparison with simulations. We observed that the profile of particle growth matched qualitatively and quantitatively with the predictions of the model. For the value of R used in these tests (R ~ 1), the model template that fits data with higher accuracy has a sticking probability close to sp=0.1. To find the best value of sp that optimizes the fit, we determined, for each of the numerical growth profiles, the rate of growth at the early stage of the process, calculated as v~N/τ, where N is the steady state number of particles in an aggregate and τ is the constant of time. Notice that N/τ is the first order of the Taylor series that approximates the curves of growth in the neighbour of t=0. Thus, using data from the model, we built a velocity of growth versus a sticking probability diagram (Figure 11b). We then used in the diagram the value of particle growth velocity determined from experiments; this allowed us to derive the best value of sticking probability that enables maximum adherence of the model to the experiments as sp=0.09. Thus, for this choice of the experimental conditions, a moderate amount of citrate in solution (R ~ 1) was simulated by a value of sp=0.09. The model and EM DLA suggest that increasing the amount of sodium citrate in solution (leading to a rise of R) has as an effect on the diminution of particle size, increases the time to the steady state and decreases the rate of reaction.

#### 3.4.7. Particle Size Distribution

We examined the ability of the model to reproduce the shape of the size distribution of particles obtained under certain experimental conditions. Figure 11e reports the size distribution of multifaceted gold particles measured at the end of the process of synthesis (i.e., approximately 120 min from growth inception), compared to the size distribution of the numerical aggregates obtained at the steady state of deposition (Figure 11e). While we cannot determine a direct equivalence between particle size and the mass of the numerical aggregates—being *inhomogeneous* variables—we can gain information on the process of growth by examining the *shapes* of the distributions. Notably, both distributions are skewed to the right, with a coefficient of skewness for the particle size distribution of about 0.8 and of ~1.1 for the distribution of the sizes of the numerical aggregates. Thus, the EM DLA simulation correctly predicted that data values are likely to cluster more towards the lower range of the dimensional scale, perhaps reflecting the fact that particle sizes cannot be less than the size of the originating seed (imposing a boundary on one side) but are not restricted by a definite upper boundary.

## 4. Conclusions

In summary, we have demonstrated the synthesis of dichroic gold sol at room temperature by an ecofriendly route. We found out that the color of the transmitted light can be easily manipulated by the addition of a third molecule to the reaction mixture. Electron microscopy and UV–Vis spectroscopy have been used exclusively for characterization. At room temperature and using a very low amount of citrate, the morphology of gold tended to be non-spherical in shape and a variety of structures were developed in the reaction mixture. This opens new insights into the reduction mechanism of gold ions by citrate molecules.

To analyze the aggregation mechanisms of the gold nanoparticles, we have developed a different formulation of classical diffusion limited aggregation (DLA) model that we have called energy minimization DLA (EM DLA) because it maintains the energy levels of the system at a lowest throughout the entire process of growth. The method, that does not have any precedent in the literature for the numerical simulation of DLA aggregates, was able to generate nanocrystals, i.e., particles with well-defined shapes, high internal correlations and high symmetries, similarly to the real particles obtained by experiments. The scheme also reproduced the size and the dynamics of growth of the gold nanoparticles.

## Data Availability

The data presented in this study are available on request from the corresponding authors.

## Supplementary Materials

The following are available online at https://www.mdpi.com/2079-4991/11/1/236/s1, Figure S1: Tyndel effect in small ≤5 nm gold nanoparticles. Figure S2: TEM images of four different types of dichroic sol., Table S1: Dominant molecular structures of citrate and aurate as function of pH., Table S2: pK_a_ of citrate acid–base equilibria., Table S3: Molecular ratios.
