# Supplementary material for: Experimental and Theoretical Studies on Sustainable Synthesis of Gold Sol Displaying Dichroic Effect
Source: Nanomaterials (Basel). 2021 Jan 18;11(1):236. doi: 10.3390/nano11010236 (PMC7830637; doi:10.3390/nano11010236)
Supplement: Supplementary file 1 [file nanomaterials-11-00236-s001.pdf]

Supporting Information

Title Experimental and Theoretical Studies on Sustainable Synthesis of Gold Sol Displaying Dichroic Effect

Anshuman Jakhmola [1,*], Raffaele Vecchione [1,2,*], Valentina Onesto [3], Francesco Gentile [4,5,*], Maurizio Celentano [1,6] and Paolo. A. Netti [1,2,7]

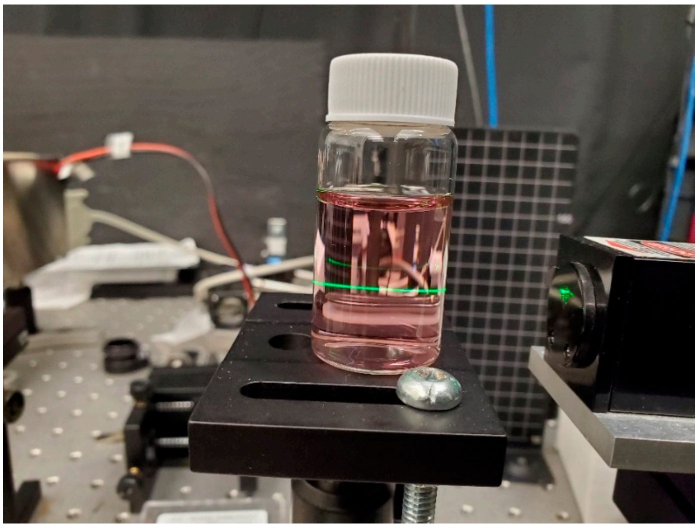

Figure S1. Tyndel effect in small ≤5 nm gold nanoparticles.

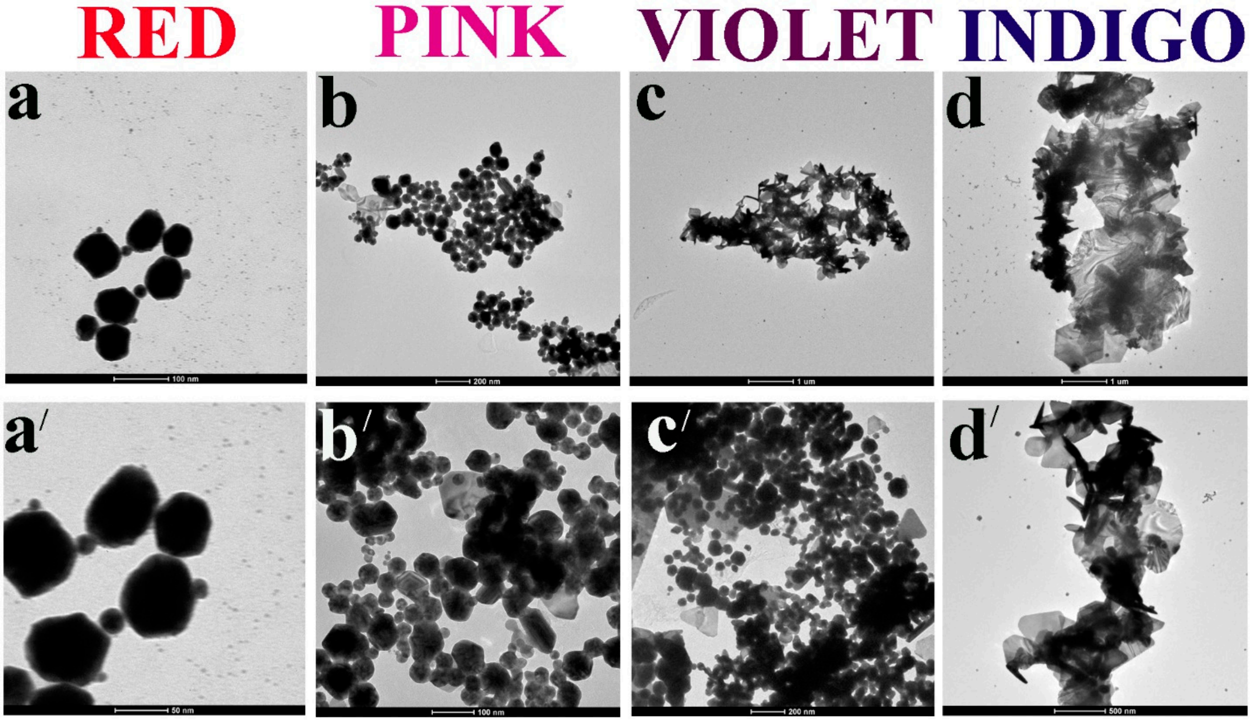

Figure S2. TEM images of four different types of dichroic sol (**a**, **a'**) Red, (**b**, **b'**) Pink, (**c**, **c'**) Violet, (**d**, **d'**) Indigo. The red sol also had small ~5-10 nm particles besides big faceted particles.

Table S1. Dominant molecular structures of citrate and aurate as function of pH.

pH	Dominant structure of citrate	Dominant structure of aurate
< 3.1	$C_6H_8O_7$	$AuCl_4^-$
3.1 – 4.8	$C_6H_7O_7^-$	$AuCl_4^-$
4.8 – 5.4	$C_6H_6O_7^{2-}$	$AuCl_4^-$
5.4 – 6.4	$C_6H_6O_7^{2-}$	$AuCl_3(OH)^-$
6.4 – 7.5	$C_6H_5O_7^{3-}$	$AuCl_2(OH)_2^-$
7.5 – 8.3	$C_6H_5O_7^{3-}$	$AuCl(OH)_3^-$
> 8.3	$C_6H_5O_7^{3-}$	$Au(OH)_4^-$

Table S2. pK_a of citrate acid-base equilibria.

Equilibrium	pK_a
$H_3C_6H_5O_7 = H^+ + H_2C_6H_5O_7^-$	3.14
$H_2C_6H_5O_7^- = H^+ + HC_6H_5O_7^{2-}$	4.76
$H_3C_6H_5O_7 = H^+ + C_6H_5O_7^{3-}$	6.40

Table S3. Molecular ratios.

Molecular Ratio (R)	pH stock trisodium citrate	pH stock chloroauric acid
0.97	8.003	2.41
